# Up-regulation of long non-coding RNA PANDAR is associated with poor prognosis and promotes tumorigenesis in bladder cancer

**DOI:** 10.1186/s13046-016-0354-7

**Published:** 2016-05-20

**Authors:** Yonghao Zhan, Junhao Lin, Yuchen Liu, Mingwei Chen, Xiaoying Chen, Chengle Zhuang, Li Liu, Wen Xu, Zhicong Chen, Anbang He, Qiaoxia Zhang, Xiaojuan Sun, Guoping Zhao, Weiren Huang

**Affiliations:** Key Laboratory of Medical Reprogramming Technology, Shenzhen Second People’s Hospital, The First Affiliated Hospital of Shenzhen University Shenzhen, Shenzhen, China; Department of Urology, Peking University First Hospital, Institute of Urology, Peking University, National Urological Cancer Center, Beijing, 100034 China; Shantou University Medical College, Shantou, 515041 China; Shanghai-MOST Key Laboratory of Health and Disease Genomics, Chinese National Human Genome Centerat Shanghai, Shanghai 200000, Shanghai, China

## Abstract

**Background:**

Long non-coding RNAs (lncRNAs) have emerged as biomarkers and important regulators of tumor development and progression. PANDAR (promoter of CDKN1A antisense DNA damage activated RNA) is a novel long non-coding RNA that acts as a potential biomarker and involves in development of multiple cancers. However, the clinical significance and molecular mechanism of PANDAR in bladder cancer is still unknown. In this study, we aimed to figure out the role of PANDAR in bladder cancer.

**Methods:**

The relative expression level of lncRNA PANDAR was determined by Real-Time qPCR in a total of 55 patients with urothelial bladder cancer and in different bladder cancer cell lines. We inhibited PANDAR expression by transfecting PANDAR specific siRNA and enhanced PANDAR expression by transfecting a PANDAR expression vector (pcDNA3.1-PANDAR). Cell proliferation was determined by using both CCK-8 assay and Edu assay. Cell apoptosis was determined by using ELISA assay, Hoechst 33342 staining and Flow cytometry. Cell migration was determined by using transwell assay. All experimental data from three independent experiments were analyzed by *χ*2 test or Student’s *t*-test and results were expressed as mean ± standard deviation.

**Results:**

We found that PANDAR was significantly up-regulated in bladder cancer tissues compared with paired-adjacent nontumorous tissues in a cohort of 55 bladder cancer patients. Moreover, increased PANDAR expression was positively correlated with higher histological grade (*P* < 0.05) and advanced TNM stage (*P* < 0.05). Further experiments demonstrated that inhibited cell proliferation/migration and induced apoptosis by silencing PANDAR were also observed in bladder cancer cells. Furthermore, over expression of PANDAR in bladder cancer cells promoted the proliferation/migration and suppressed apoptosis.

**Conclusions:**

These findings demonstrate that PANDAR plays oncogenic roles in bladder cancer and PANDAR may serve as a potential prognostic biomarker and therapeutic target of bladder cancer.

## Background

Bladder cancer is the ninth most common malignancy worldwide [1]. About 151297 newly diagnosed bladder cancer cases and 52395 bladder cancer deaths were appeared in Europe in 2014 [2]. Although in the past years there are some progresses in clinical treatment for bladder cancer, the overall survival (OS) time of bladder cancer patients has not been improved dramatically, and the 5-year survival rate for patients with bladder cancer remains at only 50–60 % [3–5]. Because the prognosis of bladder cancer is closely related to the stage of disease at diagnosis, novel diagnostic markers for early stage are urgently needed [6–9].

The long non-coding RNAs (lncRNAs) are important new members of the ncRNA family, which are longer than 200 nucleotides [10]. The rapid development of cancer genomics has highlighted the role of lncRNAs in human cancers [11–13]. Recently more and more evidences showed that lncRNAs play crucial regulatory roles in diverse biological processes, such as transcriptional regulation, cell growth and tumorigenesis [14, 15]. However, the clinical significance and molecular mechanism of lncRNAs in bladder cancer remain largely elusive. PANDAR (promoter of CDKN1A antisense DNA damage activated RNA) is a novel lncRNA that was localized at 6p21.2.

Hung et al. reported that lncRNA PANDAR was induced in a p53-dependent manner and interacts with the transcription factor NF-YA to limit the expression of pro-apoptotic genes [16]. Recently, lncRNA PANDAR originally was identified as biomarkers and was involved in development of multiple cancers [17, 18]. However, the biological function and underlying mechanism of action of lncRNA PANDAR in bladder cancer is completely unknown.

In this study, we found that lncRNA PANDAR was significantly up-regulated in bladder cancer tissue compared with paired-adjacent nontumorous tissues in a cohort of 55 bladder cancer patients. Furthermore, increased PANDAR expression was positively correlated with higher histological grade (*P* < 0.05) and advanced TNM stage (*P* < 0.05). We demonstrated that silencing PANDAR significantly inhibited proliferation/migration and induced apoptosis of the bladder cancer cells. Moreover, over expression of PANDAR in bladder cancer cells promoted the proliferation/migration and suppressed apoptosis. Our data suggest that PANDAR was a powerful tumor biomarker, which highlighted its potential clinical utility as a promising prognostic biomarker and therapeutic target.

## Results

### LncRNA PANDAR was up-regulated in bladder cancer tissues and positively correlated with higher histological grade and advanced TNM stage

**Fig. 1:**
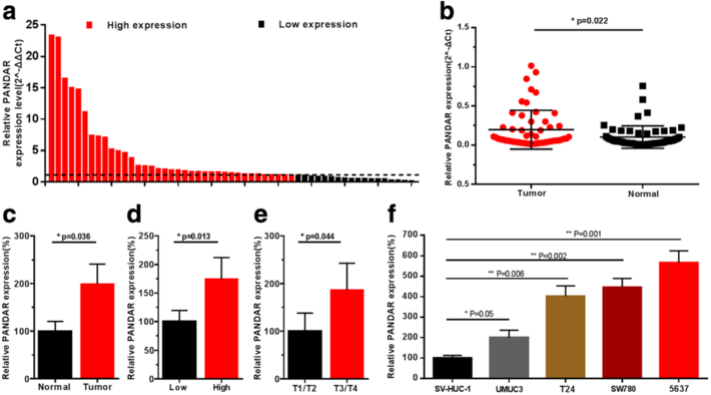
The relative expression levels of PANDAR in bladder cancer tissues and bladder cancer cell lines. The relative expression levels of PANDAR were detected using Real-Time qPCR. The heights of the columns in the chart represent the log2-transformed fold changes (bladder cancer tissue/normal bladder tissue) in PANDAR expression in 55 patients with bladder cancer (**a**). PANDAR expression levels were higher in bladder cancer tissues than that in pair-matched adjacent normal tissues (**b** and **c**). PANDAR expression was significantly higher in patients with a higher histological grade and advanced TNM stage (**d** and **e**). PANDAR was up-regulated in bladder cancer cell lines compared to normal urothelial cell line SV-HUC-1 (**f**). Data are shown as mean ± SD

The relative expression level of lncRNA PANDAR was determined by Real-Time qPCR in a total of 55 patients with urothelial bladder cancer. The lncRNA PANDAR expression fold change (bladder cancer tissue/matched normal tissue) in each patient was indicated in Fig. 1a. As shown in Fig. 1b, c, lncRNA PANDAR was up-regulated in bladder cancer tissues compared to pair-matched adjacent normal tissues. Moreover, increased PANDAR expression was positively correlated with higher histological grade and advanced TNM stage (Fig. 1d, e). These results demonstrated that lncRNA PANDAR should play oncogenic roles in bladder cancer. Respectively, clinicopathological features of patients and statistical results are shown in Tables 1 and 2.

**Table 1:** Correlation between PANDAR expression and clinicopathological features of UCB patients

Parameters Total	Group	Total	PANDAR expression		*P* value
			High	Low	
Gender	Male	40 (73 %)	27 (49 %)	13 (24 %)	0.953
	Female	15 (27 %)	10 (18 %)	5 (9 %)	
Age (years)	< 60	20 (36 %)	14 (25 %)	6 (11 %)	0.745
	≥ 60	35 (64 %)	23 (42 %)	12 (22 %)	
Tumor size (cm)	< 3 cm	21 (38 %)	11 (20 %)	10 (18 %)	0.061
	≥ 3 cm	34 (62 %)	26 (47 %)	8 (15 %)	
Multiplicity	Single	32 (58 %)	21 (38 %)	11 (20 %)	0.759
	Multiple	23 (42 %)	16 (29 %)	7 (13 %)	
Histological grade	G1	23 (42 %)	11 (20 %)	12 (22 %)	0.010*
	G2,G3	32 (58 %)	26 (47 %)	6 (11 %)	
Tumor stage T	T1,T2	38 (69 %)	22 (40 %)	16 (29 %)	0.027*
	T3,T4	17 (31 %)	15 (27 %)	2 (4 %)	
Lymph nodes metastasis	NO	53 (96 %)	16 (31 %)	36 (65 %)	0.607
	YES	2 (4 %)	1 (2 %)	1 (2 %)	

*P < 0.05 was considered significant (Chi-square test between 2 groups)

**Table 2:** Summary of clinicopathological features of tissues of bladder cancer

Pt No.	Sex	Age	Stage	Grade	Pt No.	Sex	Age	Stage	Grade
1	M	66	T2bN0M0	H	29	M	58	T4aN0M0	H
2	M	53	T1N0M0	L	30	M	63	T2aN0M0	L
3	M	75	T2bN0M0	H	31	M	50	T2bN0M0	H
4	F	64	T1N0M0	L	32	M	73	T3bN0M0	H
5	M	58	T3aN0M0	H	33	F	62	T4aN0M0	H
6	M	65	T2bN0M0	H	34	M	41	T1N0M0	L
7	F	38	T3aN0M0	H	35	M	62	T4aN0M0	H
8	M	59	T2bN0M0	H	36	M	76	T2bN0M0	L
9	M	43	T3aN0M0	H	37	M	25	T1N0M0	L
10	F	64	T2bN0M0	H	38	F	74	T3aN0M0	H
11	M	69	T1N0M0	L	39	F	70	T1N0M0	L
12	M	72	T3aN0M0	H	40	M	59	T4N0M0	H
13	F	89	T1N0M0	L	41	F	72	T1N0M0	L
14	M	68	T2bN0M0	H	42	M	46	T1N0M0	L
15	F	63	T3aN0M0	H	43	M	63	T3aN0M0	H
16	M	63	T2bN0M0	H	44	M	86	T1N0M0	L
17	M	78	T2aN0M0	L	45	M	70	T2bN0M0	H
18	M	70	T2aN0M0	L	46	M	49	T1N0M0	L
19	F	41	T2aN0M0	L	47	M	61	T3aN0M0	H
20	M	59	T2bN0M0	H	48	M	53	T2aN0M0	L
21	F	73	T2aN0M0	L	49	M	73	T2bN1M0	H
22	M	67	T2bN0M0	H	50	M	47	T2aN0M0	L
23	F	61	T3aN0M0	H	51	M	77	T3aN0M0	H
24	F	51	T1N0M0	L	52	M	66	T1N0M0	L
25	M	58	T4aN3M0	H	53	F	74	T2bN0M0	H
26	M	63	T2aN0M0	L	54	F	60	T2aN0M0	H
27	M	57	T4aN0M0	H	55	M	68	T1N0M0	L
28	M	54	T2bN0M0	H					

Pt No. patient number; M male; F female; Grade the World Health Organization 2004 classification; H high; L low; Stage the American Joint Committee on Cancer TNM classification

### LncRNA PANDAR was up-regulated in bladder cancer cell lines and specific siRNA down-regulated and pcDNA up-regulated expression of lncRNA PANDAR

**Fig. 2:**
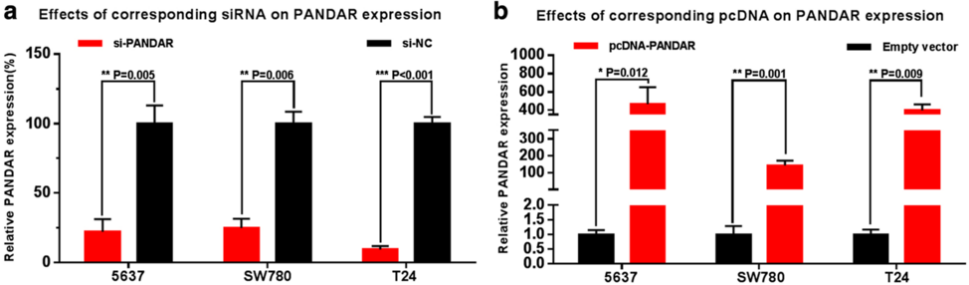
Effects of corresponding siRNA or pcDNA on PANDAR expression. The relative expression level was determined using real-time qPCR. The PANDAR specific siRNA significantly down-regulated the expression level of PANDAR in 5637, SW780, and T24 cells (**a**). The PANDAR specific pcDNA significantly up-regulated the expression level of PANDAR in 5637, SW780, and T24 cells (**b**). Data are indicated as mean ± SD

The relative expression level of PANDAR was determined by using Real-Time qPCR in different cell lines. PANDAR was up-regulated in bladder cancer cell lines compared to normal urothelial cell line SV-HUC-1 (Fig. 1f). Bladder cancer cells were cultured and then we inhibited PANDAR expression by transfecting PANDAR specific siRNA and enhanced PANDAR expression by transfecting a PANDAR expression vector (pcDNA3.1-PANDAR). At 48 h after transfection, the related expression level of PANDAR was determined by qRT-PCR and the results showed that the relative level of PANDAR in bladder cancer cells was significantly down-regulated by the PANDAR siRNA(Fig. 2a) and up-regulated by the pcDNA3.1-PANDAR (Fig. 2b).

### Silencing lncRNA PANDAR inhibited cell proliferation and overexpressing lncRNA PANDAR promoted cell proliferation

**Fig. 3:**
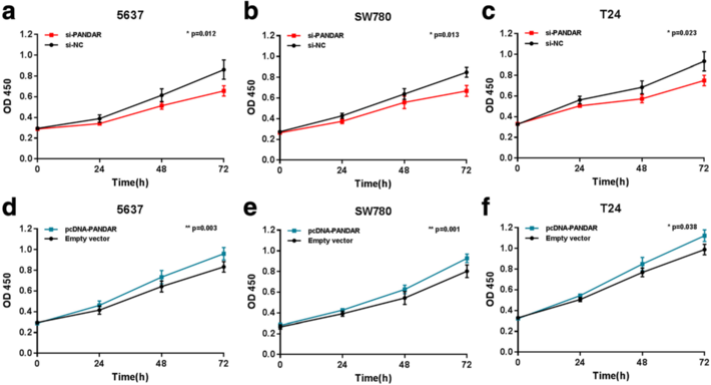
Effects of down-regulation or up-regulation of PANDAR on cell proliferation. Cell proliferation was determined by CCK-8 assay. Cell proliferation inhibition was observed in bladder cancer 5637 cells (**a**), SW780 cells (**b**) and T24 cells (**c**). Cell proliferation promotion was observed in bladder cancer 5637 cells (**d**), SW780 cells (**e**) and T24 cells (**f**). Data are shown as mean ± SD

**Fig. 4:**
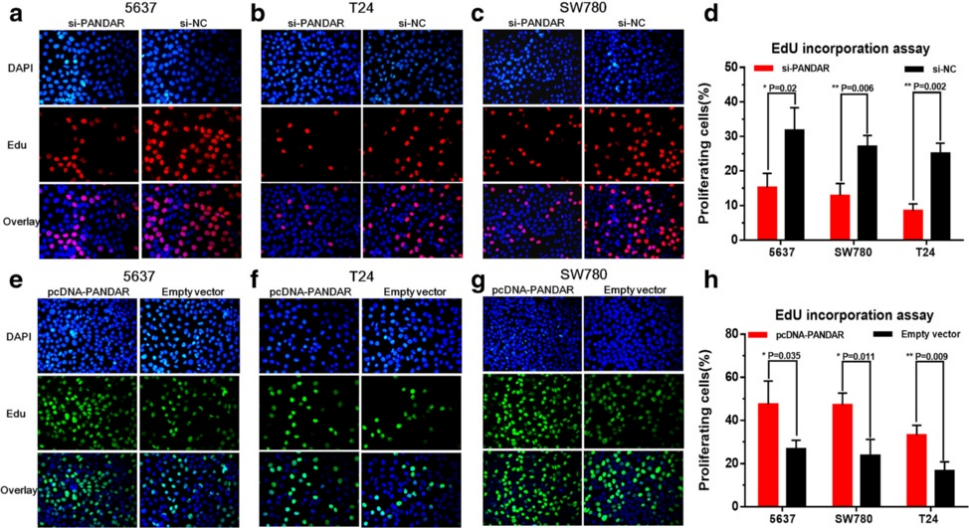
Effects of down-regulation or up-regulation of PANDAR on cell proliferation. Cell proliferation was also determined by Edu assay. Cell proliferation inhibition was observed in bladder cancer 5637 cells (**a** and **d**), SW780 cells (**b** and **d**) and T24 cells (**c** and **d**). Cell proliferation promotion was observed in bladder cancer 5637 cells (**e** and **h**), SW780 cells (**f** and **h**) and T24 cells (**g** and **h**). Data are shown as mean ± SD

We further determined whether PANDAR promotes cell proliferation in bladder cancer. Three different bladder cancer cells were transfected with PANDAR siRNA or negative control siRNA. In the same way, three different bladder cancer cells were transfected with pcDNA3.1-PANDAR or pcDNA3.1-NC and the cell proliferation changes of bladder cells were determined using both CCK-8 assay and Edu assay. Inhibited cell proliferation by silencing PANDAR was observed in 5637 cells (Fig. 3a and Fig. 4a, d), SW780 cells (Fig. 3b and Fig. 4b, d) and T24 cells (Fig. 3c and Fig. 4c, d) as expected. Promoted cell proliferation by overexpressing PANDAR was observed in 5637 cells (Fig. 3d and Fig. 4e, h), SW780 cells (Fig. 3e and Fig. 4f, h) and T24 cells (Fig. 3f and Fig. 4g, h) as expected. These results confirmed that PANDAR promotes cell proliferation in bladder cancer.

### Silencing PANDAR induced cell apoptosis and overexpressing PANDAR suppressed cell apoptosis

**Fig. 5:**
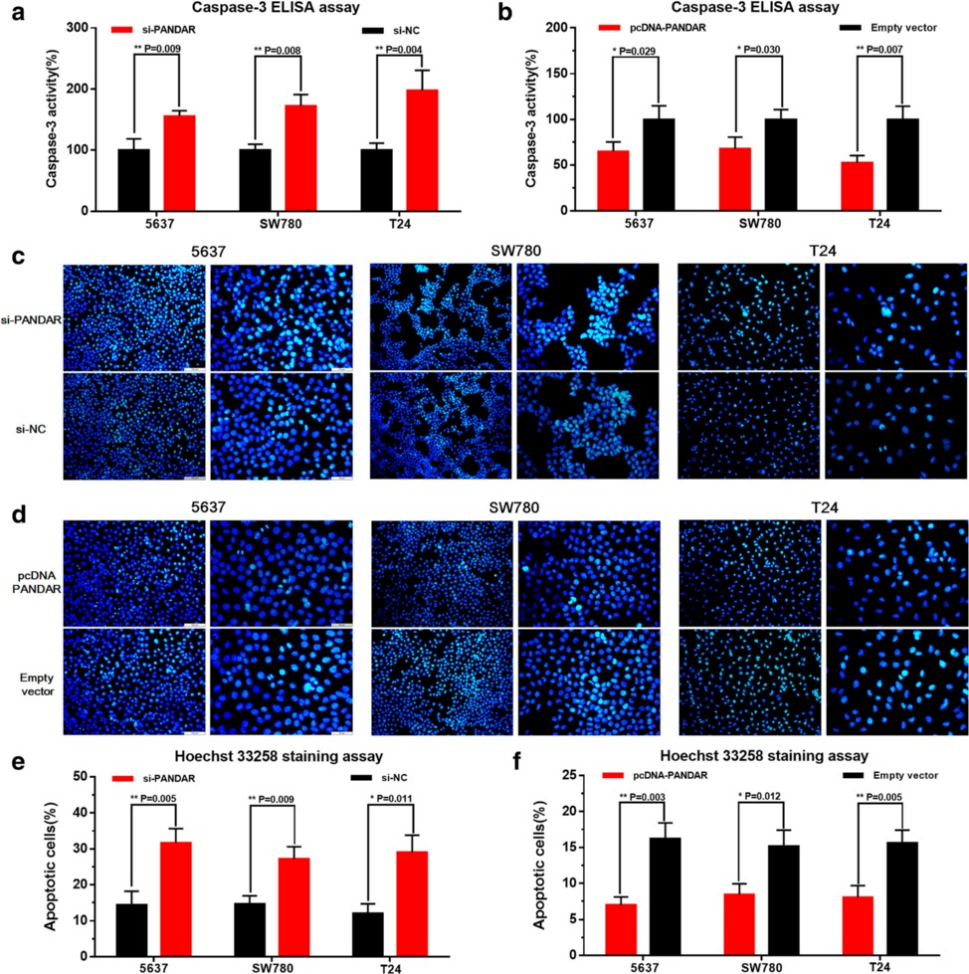
Effects of down-regulation or up-regulation of PANDAR on cell apoptosis. Cell apoptosis was determined by both ELISA assay and Hoechst 33342 staining assay. Induced cell apoptosis by silencing PANDAR was observed in bladder cancer 5637 cells, SW780 cells and T24 cells (**a**, **b** and **c**). Suppressed cell apoptosis by overexpressing PANDAR was observed in bladder cancer 5637 cells, SW780 cells and T24 cells (**d**, **e** and **f**). Data are shown as mean ± SD

**Fig. 6:**
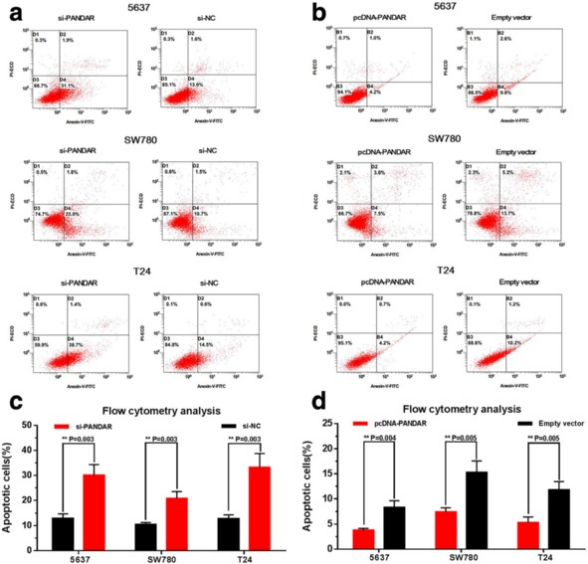
Effects of down-regulation or up-regulation of PANDAR on cell apoptosis. Cell apoptosis was also determined by flow cytometry analysis. Induced cell apoptosis by silencing PANDAR was observed in bladder cancer 5637 cells, SW780 cells and T24 cells (**a** and **c**). Suppressed cell apoptosis by overexpressing PANDAR was observed in bladder cancer 5637 cells, SW780 cells and T24 cells (**b** and **d**). Data are shown as mean ± SD

We further determined whether PANDAR suppresses cell apoptosis in bladder cancer. Three different bladder cancer cells were transfected with PANDAR siRNA or negative control siRNA. In the same way, three different bladder cancer cells were transfected with pcDNA3.1-PANDAR or pcDNA3.1-NC and the cell apoptosis changes of bladder cells were determined using ELISA assay, Hoechst 33342 staining and Flow cytometry. Induced cell apoptosis by silencing PANDAR was observed in 5637 cells, SW780 cells and T24 cells (Fig. 5a, c and Fig. 6a, c) as expected. Suppressed cell apoptosis by overexpressing PANDAR was observed in 5637 cells, SW780 cells and T24 cells (Fig. 5b, d and Fig. 6b, d) as expected. These results demonstrated that PANDAR suppresses cell apoptosis in bladder cancer.

### Silencing lncRNA PANDAR inhibited cell proliferation and overexpressing lncRNA PANDAR promoted cell migration

**Fig. 7:**
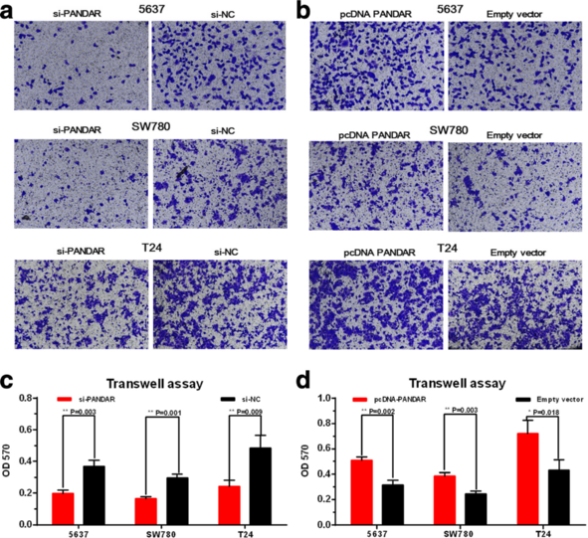
Effects of down-regulation or up-regulation of PANDAR on cell migration. Cell migration was determined by transwell assay. Inhibited cell migration by silencing PANDAR was observed in 5637, SW780 and T24 cells (**a** and **c**). Promoted cell migration by overexpressing PANDAR was observed in 5637, SW780 and T24 cells (**b** and **d**). Data are shown as mean ± SD

We further determined whether PANDAR promotes cell migration in bladder cancer. Three different bladder cancer cells were transfected with PANDAR siRNA or negative control siRNA. In the same way, three different bladder cancer cells were transfected with pcDNA3.1-PANDAR or pcDNA3.1-NC and the cell proliferation changes of bladder cells were determined using transwell assay. Inhibited cell migration by silencing PANDAR was observed in 5637, SW780 and T24 cells (Fig. 7a and c) as expected. Promoted cell migration by overexpressing PANDAR was observed in 5637, SW780 and T24 cells (Fig. 7b and d) as expected. These results demonstrated that PANDAR promotes cell migration in bladder cancer.

## Discussion

Bladder cancer is one of the most common malignancies in human populations [19]. Because at the early stage of bladder cancer there are no specific symptoms for these patents, most bladder cancers are found at advanced-stage, when treatments are less effective [20, 21]. The prognosis of bladder cancer remains quite poor, therefore, finding new prognostic biomarker and therapeutic target has enormous potential to improve the clinical strategies and outcomes of bladder cancer [22, 23].

The lncRNAs are important new members of the RNA family, which are longer than 200 nucleotides and not translated into a protein [24, 25]. Recently, numerous pieces of evidences indicate that lncRNAs play a crucial role in cancer occurrence and progression [26, 27]. As a new indentified lncRNA localized at the chromosome 6, PANDAR is 1506 nucleotides in length [28]. LncRNA PANDAR was previously reported to be up-regulated in gastric cancer. PANDAR interacts with polycomb repressive complexes (PRC1 and PRC2) and the transcription factor NF-YA to repress the transcription of senescence-promoting genes in cancer cells. However, we know nothing about the relationship between lncRNA PANDAR and bladder cancer.

To our knowledge, this is the first report of lncRNA PANDAR being involved in the development of bladder cancer. In the present study, we found that lncRNA PANDAR was significantly up-regulated in bladder cancer tissues than that in corresponding non-tumor bladder tissues. These results suggest that lncRNA PANDAR may emerge as a novel player in the state of bladder cancer. In order to understand the biological functions of lncRNA PANDAR, we detected the cell proliferation, apoptosis and migration by silencing and overexpressing lncRNA PANDAR in the related bladder cancer cell lines. Inhibited cell proliferation/migration and induced cell apoptosis by silencing PANDAR were observed in bladder cancer cells. Furthermore, overexpressing lncRNA PANDAR promoted proliferation/migration and suppressed apoptosis of the bladder cancer cells. These findings demonstrate that lncRNA PANDAR may play key roles in the progression and development in bladder cancer.

## Conclusions

In conclusion, the expression level of the lncRNA PANDAR is increased in bladder cancer tissues compared with paired-adjacent nontumorous tissues. Increased PANDAR expression has been associated with poor prognosis, likely due to the ability of PANDAR to promote cell growth and metastasis in bladder cancer cells. Cumulatively, these findings demonstrate that PANDAR plays oncogenic roles in bladder cancer and PANDAR may serve as a potential prognostic biomarker and therapeutic target of bladder cancer.

## Methods

### Patients and clinical samples collection

A total of 55 patients with urothelial carcinoma of the bladder who received partial or radical cystectomy were included in this study. Bladder cancer tissue and matched normal bladder tissue from each patient were snap-frozen in liquid nitrogen immediately after resection. Written informed consent was also obtained from all the patients. The study was approved by the Institutional Review Board of Shenzhen Second People’s Hospital, Shenzhen, China and Peking University First Hospital, Beijing, China.

### Cell lines and cell culture

Bladder cancer 5637, SW780, UMUC3, T24 and SV-HUC-1 cells used in this study were purchased from the Institute of Cell Research, Chinese Academy of Sciences, Shanghai, China. The 5637 cells and SW780 cells were cultured in RPMI-1640 Medium (Invitrogen, Carlsbad, CA, USA) plus 10 % fetal bovine serum. The UMUC3, T24 and SV-HUC-1 cells were cultured in Dulbecco’s Modified Eagle Medium (Invitrogen, Carlsbad, CA, USA) plus 10 % fetal bovine serum. Plates were then placed at 37 °C with an humidified atmosphere of 5 % CO2 in incubator.

### siRNA and pcDNA transfection

The specific small interfering RNA that targeted PANDAR (si-PANDAR) and a scrambled negative control (si-NC) were purchased from GenePharma, Shanghai, China. The expression vector (pcDNA) that express PANDAR (pcDNA-PANDAR) and a scrambled negative control (pcDNA-NC) were also purchased from GenePharma, Shanghai, China. The target sequence of si-PANDAR was 5′- GCAATCTACAACCTGTCTT -3′. The cells were cultured 24 h prior to transfection. Then, the cells were transiently transfected with corresponding si-RNA or pcDNA using Lipofectamine 2000 Transfection Reagent (Invitrogen, Carlsbad, CA, USA) according to the manufacturer’s instructions. After 48 h, cells transfected with siRNA or pcDNA were harvested for qRT-PCR.

### RNA extraction and quantitative real-time PCR

The total RNA of the tissue samples or the transfected cells were extracted using the Trizol reagent (Invitrogen, Carlsbad, CA, USA) according to the instructions. The concentration and purity of the total RNA were analyzed with UV spectrophotometer analysis at 260 nm and the electrophoresis detection showed good quality of purified RNA. cDNA was converted from total RNA by using SuperScript III® (Invitrogen) according to the manufacturer’s protocol. The primer sequences were as follows: PANDAR primers [8] forward: 5′- CTGTTAAGGTGGTGGCATTG -3′, reverse: 5′- GGAGGCTCATACTGGCTGAT -3′; GAPDH primers forward: 5′- CGCTCTCTGCTCCTCCTGTTC -3′, reverse: 5′- ATCCGTTGACTCCGACCTTCAC -3′. Quantitative real-time PCR was performed by using the ABI PRISM 7000 Fluorescent Quantitative PCR System (Applied Biosystems, Foster City, CA, USA) according to the instructions. The average value in each triplicate was used to calculate the relative amount of PANDAR using 2^−ΔΔCt methods. Experiments were repeated at least three times.

### Cell counting Kit-8 assay

Cell proliferation was determined using Cell Counting Kit-8 (Beyotime Inst Biotech, China) according to instructions. Briefly, 5 × 10^3^ cells/well were seeded in a 96-well flat-bottomed plate for 24 h, then transfected with corresponding si-RNA or pc-DNA and cultured in normal medium. At 0, 24, 48 and 72 h after transfection, 10 μl of CCK-8 (5 mg/ml) was added to each well and the cells were cultured for 1 h then determined the absorbance at a wavelength of 450 nm using an microplate reader (Bio-Rad, Hercules, CA, USA). Experiments were repeated at least three times.

### Ethynyl-2-deoxyuridine (EdU) incorporation assay

Cell proliferation was also determined by Ethynyl-2-deoxyuridine incorporation assay using an EdU Apollo DNA in vitro kit (RIBOBIO,Guangzhou, China) following the manufacturer’s instructions. Briefly, cells were incubated with 100 μl of 50 μM EdU per well for 2 h at 37 °C, at 48 h after transfected with corresponding si-RNA or pc-DNA, respectively. Then, the cells were fixed for 30 min at room temperature using 100 μl of fixing buffer (4 % polyformaldehyde containing PBS). Subsequently, the cells were incubated with 50 μl of 2 mg/ml glycine for 5 min followed by washing with 100 μl of PBS. After permeabilization with 0.5 % TritonX, the cells were reacted with 1X Apollo solution for 30 min at room temperature in the dark. After that, cells were incubated with 100 μl of 1X Hoechst 33342 solution for 30 min at room temperature in the dark followed by washing with 100 μl of PBS. The cells were then visualized under a fluorescence microscopy. Experiments were repeated at least three times.

### Cleaved Caspase-3 ELISA assay

Cell apoptosis was determined by ELISA assay. Briefly, 5 × 10^5^ cells/well were seeded in a 6-well plate for 24 h, then transfected with corresponding si-RNA or pc-DNA, respectively. At 48 h after transfection, Cell cleaved caspase-3 activity was measured using the Caspase-3 Colorimetric Assay kit (Abcam, Cambridge, UK) according to the manufacturer’s protocol. Experiments were repeated at least three times in duplicates.

### Hoechst 33342 staining assay

Cell apoptosis was also determined by Hoechst 33258 staining assay. At 48 h after transfection with corresponding si-RNA or pc-DNA, apoptotic cells were also observed by using the Hoechst 33258 staining kit (Life, Eugene, OR, USA) according to the manufacturer’s instructions. Experiments were repeated at least three times.

### Flow cytometry analysis of cell apoptosis

Cells were cultured in normal medium and transfected with corresponding si-RNA or pc-DNA. Cells were collected after transfection for 48 h, and the translocation of phosphatidylserine in treated cells was detected using the Annexin-V-FLUOS staining kit (Roche Applied Science, Mannheim, Germany). Briefly, after being labeled with 5 μl of annexin V-FITC and 2 μl propidium iodide (PI), cells were suspended in 500 μl of binding buffer and incubated at room temperature in the dark for 15 min. Cell apoptosis was then determined by using flow cytometry (EPICS, XL-4, Beckman, CA, USA). Experiments were repeated at least three times.

### Transwell assay

The cell motility assay were also performed using a transwell insert (8 μm, Corning). Cells were cultured in normal medium and transfected with corresponding si-RNA or pc-DNA. 24 h after transfection, 5x104 cells were first starved in 200 ml serumfree medium and then placed in the uncoated dishes. The lower chamber was filled with 500 ml of complete medium. The cells were incubated for 48 h at 37 °C, and then the cells that had migrated to the bottom surface of the filter membrane were stained with 0.5 % crystal violet solution and photographed in five preset fields per insert. Then the absorbance were determined at a wavelength of 570 nm using an microplate reader (Bio-Rad, Hercules, CA, USA). The results represented the average of three independent experiments.

### Statistical analyses

All experimental data from three independent experiments were analyzed by *χ*2 test or Student’s *t*-test and results were expressed as mean ± standard deviation. *P*-values of less than 0.05 were considered to be statistically significant. All statistical tests were conducted by SPSS version 19.0 software (SPSS Inc. Chicago, IL, USA).

## References

[1] Witjes JA, Compérat E, Cowan NC (2014). EAU guidelines on muscle-invasive and metastatic bladder cancer: summary of the 2013 guidelines. Eur Urol.

[2] Siegel R, Ma J, Zou Z (2014). Cancer statistics, 2014[J]. CA Cancer J Clin.

[3] Marta GN, Hanna SA, Gadia R (2012). The role of radiotherapy in urinary bladder cancer: current status. International Braz J Urol.

[4] Racioppi M, D’Agostino D, Totaro A (2011). Value of current chemotherapy and surgery in advanced and metastatic bladder cancer. Urol Int.

[5] Chen J, Wang L, Tang Y (2016). Maspin enhances cisplatin chemosensitivity in bladder cancer T24 and 5637 cells and correlates with prognosis of muscle-invasive bladder cancer patients receiving cisplatin based neoadjuvant chemotherapy. J Exp Clin Cancer Res.

[6] Lewis JD, Ferrara A, Peng T (2011). Risk of bladder cancer among diabetic patients treated with pioglitazone interim report of a longitudinal cohort study. Diabetes Care.

[7] Zhan Y, Liu Y, Lin J (2015). Synthetic Tet-inducible artificial microRNAs targeting β-catenin or HIF-1α inhibit malignant phenotypes of bladder cancer cells T24 and 5637. Sci Rep.

[8] Wang C, Ge Q, Zhang Q (2016). Targeted p53 activation by saRNA suppresses human bladder cancer cells growth and metastasis. J Exp Clin Cancer Res.

[9] Zhuang CL, Fu X, Liu L (2015). Synthetic miRNA sponges driven by mutant hTERT promoter selectively inhibit the progression of bladder cancer. Tumor Biol.

[10] Gutschner T, Diederichs S (2012). The hallmarks of cancer: a long non-coding RNA point of view. RNA Biol.

[11] Tsai MC, Manor O, Wan Y (2010). Long noncoding RNA as modular scaffold of histone modification complexes. Science.

[12] Lin J, Liu Y, Zhan Y (2016). Synthetic Tet-inducible small hairpin RNAs targeting hTERT or Bcl-2 inhibit malignant phenotypes of bladder cancer T24 and 5637 cells. Tumour Biol.

[13] Wapinski O, Chang HY (2011). Long noncoding RNAs and human disease. Trends Cell Biol.

[14] Liu Y, Zeng Y, Liu L (2014). Synthesizing AND gate genetic circuits based on CRISPR-Cas9 for identification of bladder cancer cells. Nat Commun.

[15] Wang KC, Yang YW, Liu B (2011). A long noncoding RNA maintains active chromatin to coordinate homeotic gene expression. Nature.

[16] Guttman M, Rinn JL (2012). Modular regulatory principles of large non-coding RNAs. Nature.

[17] Zhuang C, Li J, Liu Y (2015). Tetracycline-inducible shRNA targeting long non-coding RNA PVT1 inhibits cell growth and induces apoptosis in bladder cancer cells. Oncotarget.

[18] Ma P, Xu T, Huang M (2016). Increased expression of LncRNA PANDAR predicts a poor prognosis in gastric cancer. Biomed Pharmacother.

[19] Siegel R, Naishadham D, Jemal A (2012). Cancer statistics, 2012. CA Cancer J Clin.

[20] Stenzl A, Cowan NC, De Santis M (2011). Treatment of muscle-invasive and metastatic bladder cancer: update of the EAU guidelines. Eur Urol.

[21] James ND, Hussain SA, Hall E (2012). Radiotherapy with or without chemotherapy in muscle-invasive bladder cancer. N Engl J Med.

[22] Suriano F, Santini D, Perrone G (2013). Tumor associated macrophages polarization dictates the efficacy of BCG instillation in non-muscle invasive urothelial bladder cancer. J Exp Clin Cancer Res.

[23] Burger M, Catto JWF, Dalbagni G (2013). Epidemiology and risk factors of urothelial bladder cancer. Eur Urol.

[24] Zhou J, Zhi X, Wang L (2015). Linc00152 promotes proliferation in gastric cancer through the EGFR-dependent pathway. J Exp Clin Cancer Res.

[25] Amaral PP, Clark MB, Gascoigne DK (2011). lncRNAdb: a reference database for long noncoding RNAs. Nucleic Acids Res.

[26] Liu L, Liu Y, Zhang T (2016). Synthetic Bax-Anti Bcl2 combination module actuated by super artificial hTERT promoter selectively inhibits malignant phenotypes of bladder cancer. J Exp Clin Cancer Res.

[27] Yang L, Lin C, Jin C (2013). lncRNA-dependent mechanisms of androgen-receptor-regulated gene activation programs. Nature.

[28] Puvvula PK, Desetty RD, Pineau P (2014). Long noncoding RNA PANDAR and scaffold-attachment-factor SAFA control senescence entry and exit. Nat Commun.

