# Correction: Up-regulation of long non-coding RNA PANDAR is associated with poor prognosis and promotes tumorigenesis in bladder cancer

**DOI:** 10.1186/s13046-023-02773-3

**Published:** 2023-07-29

**Authors:** Yonghao Zhan, Junhao Lin, Yuchen Liu, Mingwei Chen, Xiaoying Chen, Chengle Zhuang, Li Liu, Wen Xu, Zhicong Chen, Anbang He, Qiaoxia Zhang, Xiaojuan Sun, Guoping Zhao, Weiren Huang

**Affiliations:** 1 Key Laboratory of Medical Reprogramming Technology, Shenzhen Second People’s Hospital, The First Affiliated Hospital of Shenzhen University Shenzhen, Shenzhen, China; 2 Department of Urology, Institute of Urology, Peking University First Hospital, Peking University, National Urological Cancer Center, Beijing, 100034 China; 3 Shantou University Medical College, Shantou, 515041 China; 4 Shanghai-MOST Key Laboratory of Health and Disease Genomics, Chinese National Human Genome Center at Shanghai, Shanghai, 200000 China

**Correction:** ***J Exp Clin Cancer Res*** **35, 83 (2016)**

10.1186/s13046-016-0354-7

Following publication of the original article [1], an error was identified in Figure 5 and Table 1, specifically:

- Figure 5c - the Hoechst image of 5637 si-PANDAR group was misplaced
- Table 1 - the number of high and low expression in no lymph nodes metastasis were inadvertently misplaced, and the statistical significance remained unchanged after reanalyzing the data of Table 1

The corrected Figure 5 and Table 1 are given here. The correction does not affect the conclusions of the article.

**Fig. 5:**
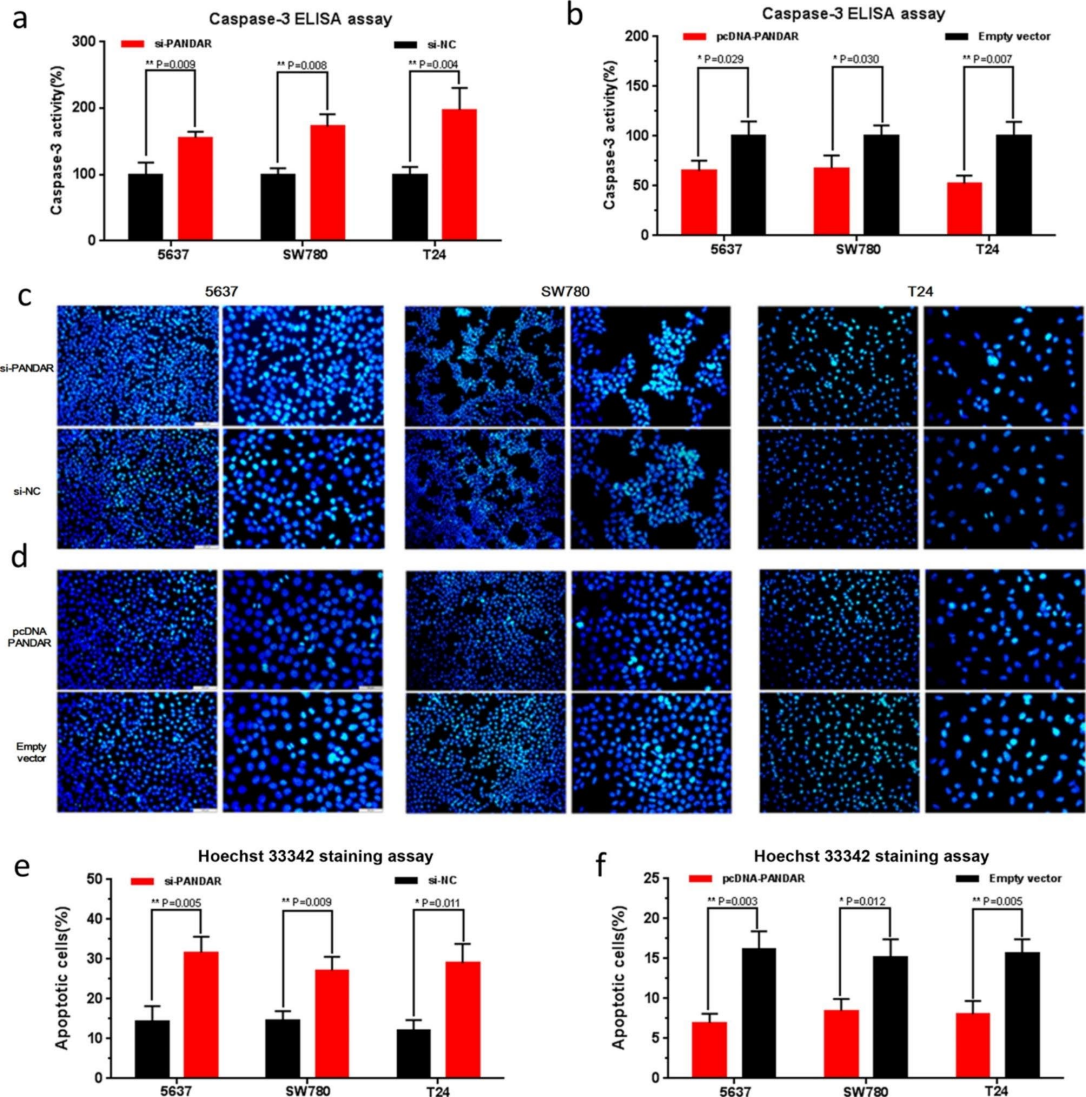
Effects of down-regulation or up-regulation of PANDAR on cell apoptosis. Cell apoptosis was determined by both ELISA assay and Hoechst 33,342 staining assay. Induced cell apoptosis by silencing PANDAR was observed in bladder cancer 5637 cells, SW780 cells and T24 cells (**a, b** and **c**). Suppressed cell apoptosis by overexpressing PANDAR was observed in bladder cancer 5637 cells, SW780 cells and T24 cells (**d, e** and **f**). Data are shown as mean ± SD

**Table 1:** Correlation between PANDAR expression and clinicopathological features of UCB patients

Parameters Total	Group	Total	PANDAR expression		*p* value
			High	Low	
Gender	Male	40(73%)	27(49%)	13(24%)	1.000
	Female	15(27%)	10(18%)	5(9%)	
Age (years)	< 60	20(36%)	14(25%)	6(11%)	0.745
	≥ 60	35(64%)	23(42%)	12(22%)	
Tumor size (cm)	< 3 cm	21(38%)	11(20%)	10(18%)	0.064
	≥ 3 cm	34(62%)	26(47%)	8(15%)	
Multiplicity	Single	32(58%)	21(38%)	11(20%)	0.759
	Multiple	23(42%)	16(29%)	7(13%)	
Histological grade	L	23(42%)	11(20%)	12(22%)	0.009 *
	H	32(58%)	26(47%)	6(11%)	
Tumor stage T	T1,T2	38(69%)	22(40%)	16(29%)	0.027 *
	T3,T4	17(31%)	15(27%)	2(4%)	
Lymph nodes metastasis	NO	53(96%)	36(65%)	17(31%)	1.000
	YES	2(4%)	1(2%)	1(2%)	

*p < 0.05 was considered significant (Chi-square test between 2 groups).
